# Case Report: Solitary mastocytoma treated successfully with topical tacrolimus

**DOI:** 10.12688/f1000research.3253.1

**Published:** 2014-08-01

**Authors:** M. S. Sukesh, Ameet Dandale, Rachita Dhurat, Ankur Sarkate, Smita Ghate

**Affiliations:** 1 Dermatology Department, LTM Medical College and General Hospital, Sion, Mumbai, 400022, India

## Abstract

Solitary mastocytoma, a rare dermatological entity accounts for 10-15% of cutaneous mastocytosis. We report a rare case of solitary mastocytoma presenting at birth, treated successfully with topical tacrolimus. Along with reassurance and strict avoidance of triggering factors, no recurrence was reported within the one year follow-up period.

## Introduction

Solitary mastocytoma, a rare dermatological entity, represents the second most common type of cutaneous mastocytoma. Solitary mastocytomas constitute 10–20% of all childhood cutaneous mastocytosis. They usually present within 2 years of age, mostly within first 3 months ^1^.

We report a case of solitary mastocytoma presenting a birth that was treated successfully with topical tacrolimus with no recurrences noted during a one year follow-up period.

## Case report

**Figure 1.**
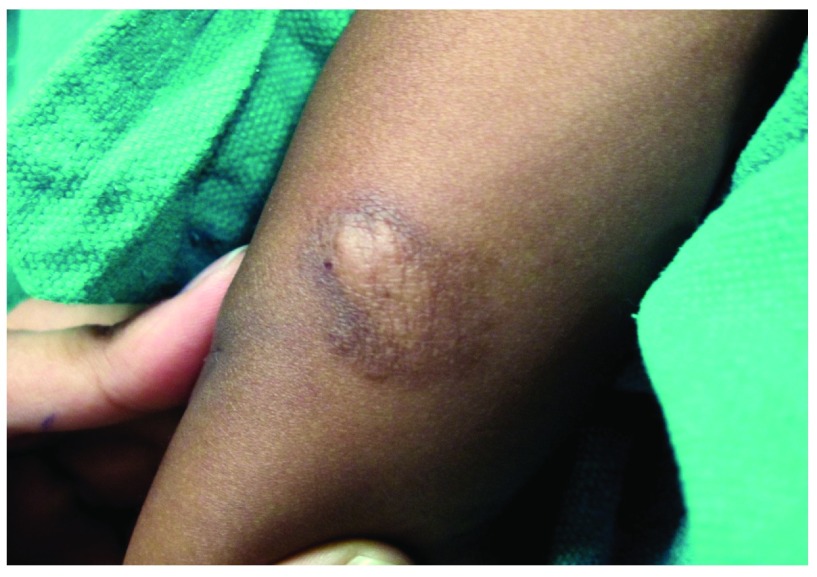
A solitary, 3.5 × 6.5 cm, non-tender, minimally elevated plaque with edematous central shiny skin made more apparent on scratching the lesion with the blunt end of a pin (positive Darier’s sign) with peripheral marginal hyperpigmentation over the left elbow.

**Figure 2.**
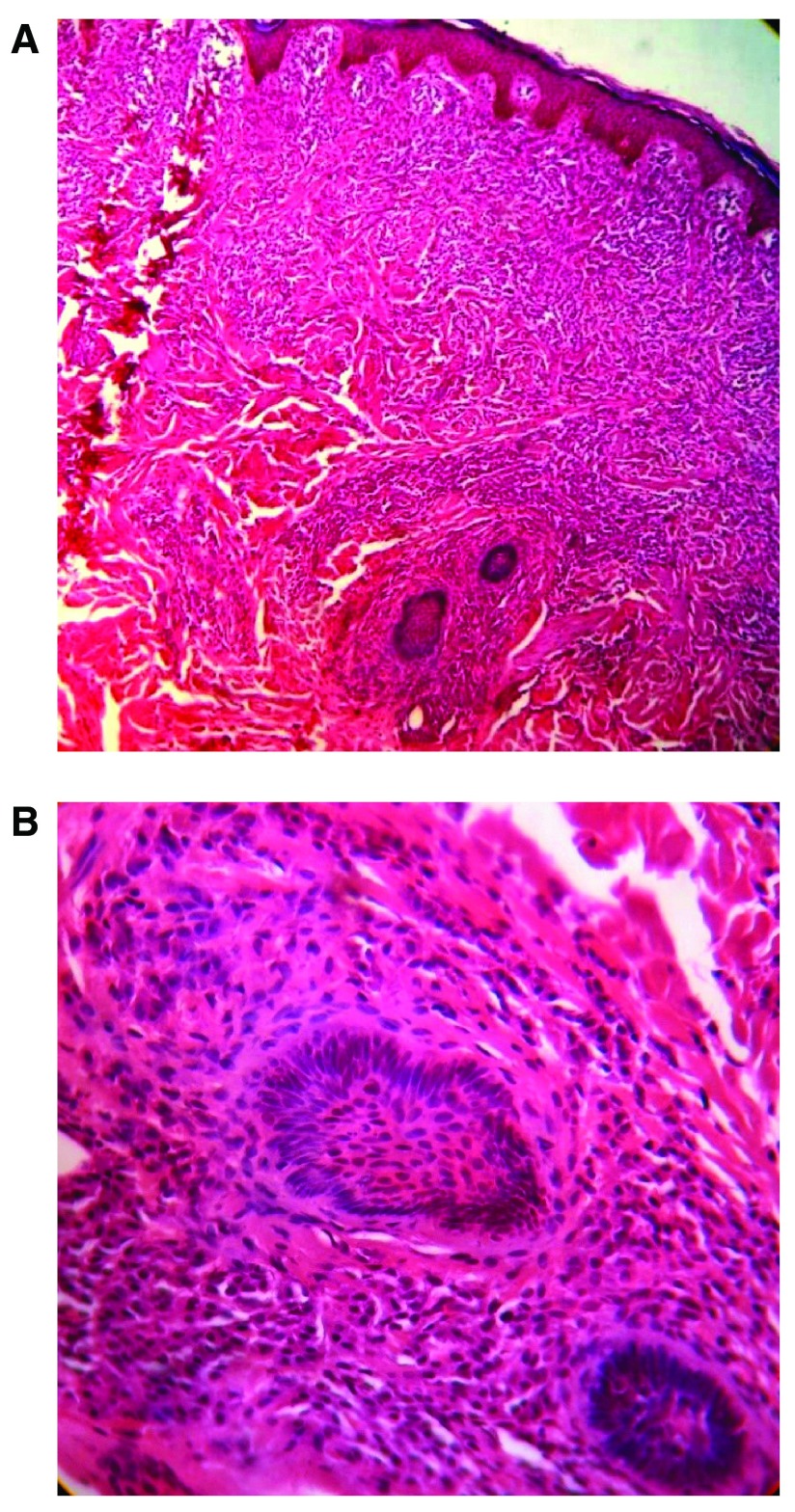
**a**, Dense monomorphic inflammatory infiltrate in upper and mid dermis; **b**, Dense monomorphic inflammatory infiltrate consisting of round to oval cells with clear cytoplasm noted at 40× magnification.

**Figure 3.**
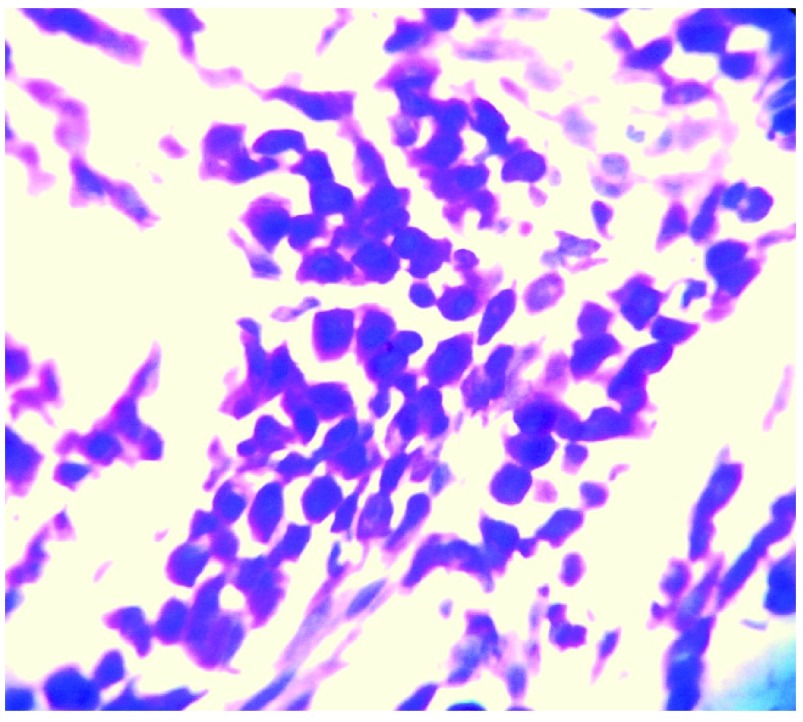
Metachromatic staining of the monomorphic mast cells with Toluidine blue staining.

An eighteen month old girl presented with a solitary, itchy dark coloured, minimally elevated lesion over her left elbow that had been evident since birth. The lesion used to itch and swell on scratching, bathing and toweling of the area. The child was otherwise healthy and no other systemic manifestations were noted. Clinical examination revealed a solitary, 3.5 × 6.5 cm, non-tender, minimally elevated plaque with central shiny skin and peripheral marginal hyperpigmentation over left elbow. On scratching the lesion with the blunt end of a pin, the central shiny skin became edematous and itchy (positive Darier’s sign) ( Figure 1). Hematological and biochemical investigations were within normal limits. A 5 mm biopsy of the skin tissue obtained from the center of the lesion revealed a dense monomorphic inflammatory infiltrate consisting of round to oval cells with clear cytoplasm and centrally located nuclei in the upper and mid dermis ( Figure 2a, 2b). Special staining with toluidine blue revealed metachromatic staining of the monomorphic mast cells, confirming the diagnosis of mastocytoma ( Figure 3).

**Figure 4.**
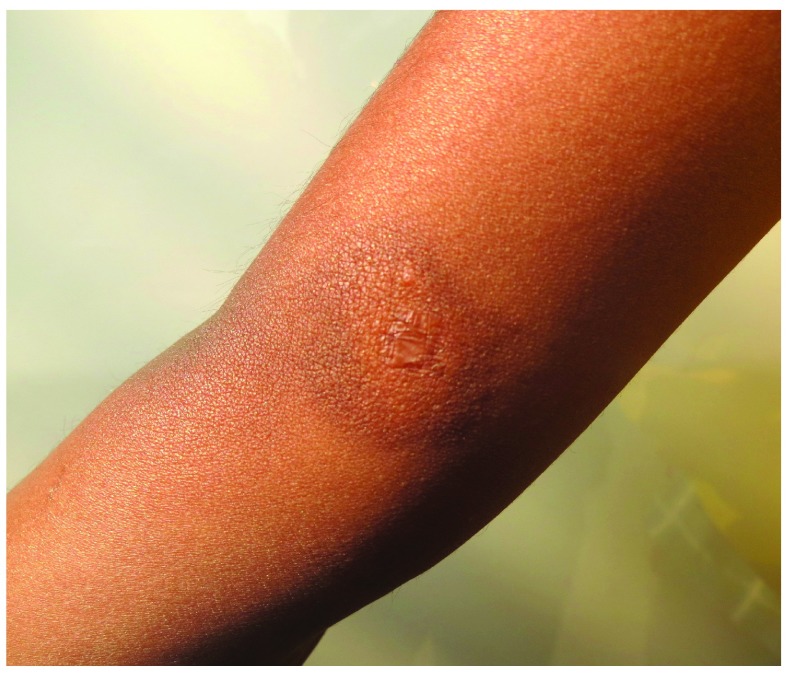
Complete subsidence of the lesion with residual marginal pigmentation noted at the end of three months of therapy. The central atrophic scar due to biopsy can be seen in the centre of the lesion.

The child was treated with topical tacrolimus 0.03% ointment which was applied on the lesion site twice daily. The child was also prescribed an oral antihistamine (levocetirizine syrup, 1.25 mg once a day). By the end of third month, complete subsidence of the lesion was noticed with residual hyperpigmentation, negative Darier's sign, and no signs of atrophy. This treatment was continued for another four months which led to resolution of the lesion with residual hyperpigmentation, negative Darier’s sign, and no signs of atrophy. Treatment was continued with only a once a day application of topical tacrolimus for a month after clinical resolution to prevent further recurrence ( Figure 4). Reassurance and strict avoidance of triggering factors such as pressure, friction (rubbing or toweling of the lesion), extreme temperature changes, intake of mast cell degranulating agents like aspirin, NSAIDS, morphine, codeine (especially in the form of cough preparations) has led to no recurrence of the child’s symptoms during a 1 year follow-up period.

## Discussion

Solitary mastocytoma, the second most common type of cutaneous mastocytosis, accounts for 10–15% of cutaneous mastocytosis ^1^. Nearly half of solitary mastocytomas present within the first 3 months of life and the remaining half during the first year ^2^. Solitary mastocytoma presenting in adults has also been noted ^3^. The most common locations of mastocytomas are on the trunk, neck, and arms.

Most solitary mastocytomas are about 1–5 cm in diameter and are seen as skin areas that are colored yellow to brown and present as minimally elevated plaques with a smooth shiny surface having a soft to rubbery consistency. The lesion turns edematous and itchy on manipulation [rubbing or trauma to the lesion]. Mild tenderness and the formation of vesicles or bulla can also occur ^4^. These features can sometimes be so mild that they may not come to the attention of parents.

Diagnosis is by biopsy that reveals a dense monomorphic inflammatory infiltrate consisting of round to oval mast cells containing a clear cytoplasm and centrally located nuclei in the dermis. Confirmation of diagnosis is usually by special staining with toluidine blue that reveals the metachromatic staining of the monomorphic mast cells ^5^.

The course of solitary mastocytomas is benign and the disease is self-limited. Systemic involvement is uncommon and complete spontaneous resolution is expected in months to years’ time. Reassurance along with avoidance of triggering factors such as pressure, friction (rubbing or toweling of the lesion), physical exertion, extreme temperature changes, emotional stress, intake of mast cell degranulating agents like aspirin, NSAIDS, morphine, codeine (particularly in cough preparations), alcohol and radio contrast dyes are of utmost importance ^6^.

In symptomatic patients, oral H1 and H2 antihistamines are commonly used. Topical steroids with or without occlusion, intralesional steroids, oral sodium cromoglycate, oral ketotifen and surgical excision are other treatment options ^6, 7^. Though topical steroids have shown good results, their topical and systemic side effects are a matter of concern, especially when treating infants.

Tacrolimus and pimecrolimus are topical immunomodulators, the first in a new class of topical calcineurin inhibitors. These drugs act as immunosuppressants by binding to a cytosolic ligand in the cytoplasm of T cells called FK506-binding protein (FKBP) and inhibit the cytoplasmic enzyme calcineurin, thus inhibiting the activation and maturation of T cells and blocking transcriptional activation of several cytokine genes – interleukin (IL)-2 [mainly], IL-4, IL-10, interferon-γ, tumor necrosis factor-α, and granulocyte–macrophage colony-stimulating factor ^8^.

Other immunomodulatory effects of tacrolimus include the inhibition of mast cell adhesion and the inhibition of the release of mediators from mast cells and basophils ^9^, which might explain its efficacy in the improvement of the lesion and alleviation of the symptoms in cutaneous mastocytosis.

These immunomodulators offer advantages over corticosteroids in terms of a more selective action, no associated systemic side-effects, and the absence of associated skin atrophy, depigmentation and telangiectasia.

This case report demonstrates that topical calcineurin inhibitors can be considered as a safe and efficacious modality of treatment in cutaneous mastocytoma.

## Consent

Written informed consent for publication of the clinical details and clinical images was obtained from the father of the patient.
