# Supplementary material for: Case Report: Solitary mastocytoma treated successfully with topical tacrolimus
Source: F1000Res. 2014 Aug 1;3:181. [Version 1] doi: 10.12688/f1000research.3253.1 (PMC4156028; doi:10.12688/f1000research.3253.1)
Supplement: Peer review history

# Peer review: Referee response for version 1

**DOI:** 10.5256/f1000research.3493.r5872

**Published:** 2014-08-19

**Authors:** Regina Fölster-Holst

**Affiliations:** 1 Dermatologische Klinik, Universität Kiel, Kiel, Germany

The authors described an 18-mo old girl with a solitary mastocytoma, which was successfully treated with tacrolimus. This might suggest that the mastocytoma requires therapy. This is not the case. Mastocytomas are self-limited and usually don´t need therapy. The most important management is the avoidance of known trigger factors. It is not clear whether the reduction is due to the self-limiting nature of the tumor or to the therapy.

Pathogenetically and from the mechanism of action of tacrolimus, which prevents mast cell degranulation, an improvement of mastocytoma can be expected. However it should be made clear that the indication for treatment of a solitary mastozytoma should be made very cautiously.

I have read this submission. I believe that I have an appropriate level of expertise to confirm that it is of an acceptable scientific standard.

# Peer review: Referee response for version 1

**DOI:** 10.5256/f1000research.3493.r5676

**Published:** 2014-08-19

**Authors:** Robert Sidbury

**Affiliations:** 1 Dermatology Division, Seattle Children's Hospital, Seattle, WA, USA

I thank the authors for this interesting article.

I believe it is worthy of indexation because there is surprisingly little in the literature about this alternative treatment. I might make a few small suggestions for consideration:

1. Is solitary mastocytoma really "rare"?  I see an awful lot of them for such a designation even taking into consideration referral bias. Uncommon might be a better descriptor.
2. Could the line " *Diagnosis is by biopsy...*" be modified?  I do not think these absolutely must be biopsied and in fact almost never do. If there is a + Dariers sign and a strong clinical suspicion this presentation is specific enough that I do not think biopsy is mandatory. As a pediatric dermatologist I do all I can to avoid biopsying when not absolutely necessary and I worry readers might take this line to imply diagnosis mandatory for diagnosis of mastocytoma. It is not.
3. Can we still call tacrolimus and pimecrolimus "new" given they have been available almost 15 years now?
4. I think in fairness the authors must mention the boxed warning about this class of medications somewhere. If the authors cite the concerns for topical and systemic side effects of topical steroids as they do I think they must balance this by mentioning the biggest barrier to using these agents - the black box.
5. I might have included two references the authors omitted:

- Correia O, Duarte AF, Quirino P *et al.*: Cutaneous mastocytosis: Two pediatric cases treated with topical pimecrolimus. *Dermatology Online Journal. *2010; **16**(5): 8
  This describes two cases similar to the authors' treated successfully with pimecrolimus
- Avshalumov K, Pichardo R, Jorizzo JL *et al.*: Bullous mastocytosis: report of a patient and a brief review of the literature. *Am J Dermatopath.* 2008; **30**(5):455-7
  Cutaneous mastocytosis - albeit not mastocytoma - treated with a number of things including topical tacrolimus.

I have read this submission. I believe that I have an appropriate level of expertise to confirm that it is of an acceptable scientific standard, however I have significant reservations, as outlined above.

# Peer review: Referee response for version 1

**DOI:** 10.5256/f1000research.3493.r5674

**Published:** 2014-08-05

**Authors:** Mohamed Badawy Abdel-Naser

**Affiliations:** 1 Faculty of Medicine, Ain Shams University, Cairo, Egypt

- In the abstract and introduction sections the authors stated that solitary mastocytoma accounts for 10-15% of cutaneous mastocytosis whereas in the introduction section it was 10-20%.
- The lesion was present since birth, but parents sought medical advice after 18 months. Was there any particular reason for this delay? In particular, was any other previous treatment(s) prescribed for the child?
- The magnification of Fig, 2 was mentioned as 40X but it seems that Fig. 2A and 2B have different magnifications. Authors should also insert the magnification of Fig.3.
- As per any case report describing a treatment for a disorder known to be self-limited, it is uncertain whether the resolution is due to the applied drug or due to natural spontaneous subsidence.

I have read this submission. I believe that I have an appropriate level of expertise to confirm that it is of an acceptable scientific standard.
